# The influence of teaching variables in the educational processes of students with rare diseases

**DOI:** 10.3389/fpsyg.2022.1046643

**Published:** 2023-01-19

**Authors:** Ramón García-Perales, Ascensión Palomares-Ruiz, Eduardo García-Toledano, María Inés Martín-García

**Affiliations:** Faculty of Education, Department of Pedagogy, University of Castilla-La Mancha (UCLM), Albacete, Spain

**Keywords:** teaching, rare diseases, knowledge, perceptions, inclusive education

## Abstract

**Introduction:**

Teachers have a key role in their students’ educational inclusion processes. Numerous variables influence their professional work and determine how they approach teaching.

**Methods:**

In this study, 574 teachers teaching non-university educational stages in the Autonomous Community of Castilla-La Mancha were queried about their knowledge and perceptions regarding students with rare diseases, examining the extent to which there were personal variables that could have an impact on that.

**Results:**

The results indicate the need to expand training to increase levels of knowledge in the educational arena about rare diseases, especially about how they are conceptually described and their categorization and prevalence. All of the teaching variables evaluated were statistically significant, with p < 0.001 in most of the elements evaluated according to each of the following variables: sex, age, job position, teaching experience, and higher qualifications. This shows that there are teaching variables that influence the inclusion of students with rare diseases. Being aware of them should be a priority in order to increase teacher empowerment for the delivery of inclusive educational processes.

**Discussion:**

All students exhibit distinctive characteristics and teachers play an essential role in offering them quality individualized education. The full inclusion of all students is something educational systems have yet to achieve, and this study aimed to contribute to that goal, in this case for schoolchildren with rare diseases.

## 1. Introduction

Teachers play a key role in the teaching and learning processes in all educational dimensions. This means that how they teach is also fundamental to their students’ educational and social inclusion (Colmenero et al., 2014; Ewing et al., 2018; Arnaiz et al., 2020). To achieve this inclusion, teachers have to offer educational responses tailored to each student’s characteristics and potential (Palomares-Ruiz and García-Perales, 2020). Studies have backed up the importance of teachers’ roles in their students’ inclusion (González-Gil et al., 2019; Pérez-Gutiérrez et al., 2021; Ginevra et al., 2022).

Many studies have reported that teachers’ have favorable attitudes toward the inclusion of students with educational needs (Álvarez-Castillo and Buenestado-Fernández, 2015; Sharma and Jacobs, 2016; Polo and Aparicio, 2018; Saloviita, 2020), but also that they demand greater teacher training to address the heterogeneity of educational needs in their classrooms (González-Gil et al., 2019; Florian and Camedda, 2020; Falla et al., 2022). School teaching staff includes professionals whose work is to meet students’ educational needs, working with other teachers and families in inclusion (Nilsen et al., 2019; Sundqvist and Hannås, 2021). It is essential for all education professionals to participate to achieve the goal of inclusion, which will facilitate progress toward a social transformation that will become a reality from an inclusive perspective (Nilsen, 2017).

In recent years, there have been innovations and changes to progressively move toward inclusive education (Meijer and Watkins, 2019; García-Peñalvo et al., 2022; Jia et al., 2022). These have included including students with educational needs in ordinary schools, increased teacher training, the spread of educational professionals dedicated to dealing with students with educational needs, increased and updated teaching resources, the use of information and communication technologies in educational processes, the variety of groupings in the classroom, and the management of specific methodologies. Despite this, there is still a long way to go to achieve fully inclusive teaching and learning processes (Nilsen et al., 2019; Sundqvist and Hannås, 2021). The breakneck pace of change in culture and society throws up new challenges and obstacles to achieving inclusive educational systems. Considering the teaching work to promote educational inclusion and in order to promote the general spread of quality inclusive educational processes for all students, in this study, we address the educational inclusion of students with rare diseases from the perspective of the teacher. This is a subject that deserves special consideration due to the scarcity of existing research (García-Perales et al., 2022).

Students with rare diseases are defined as those who suffer from a disease with a low prevalence, below 100 cases per 100,000 people (Charco et al., 2021). The large number and variety of different conditions (Richter et al., 2015; Nguengang-Wakap et al., 2020; Orphanet, 2022) make it hard to deal with them in the educational field. There are estimated to be more than 7,000 rare diseases, with up to 88.1% of them in Spain being diagnosed in childhood (Ahedo et al., 2021), representing approximately 1.8% of the total Spanish population (Federación Española de Enfermedades Raras, 2018). The low prevalence, the variety, and the time taken to identify diseases (Gainotti et al., 2018; Vandeborne et al., 2019), among other aspects, result in a lack of knowledge and deficiencies in how they are addressed socially, educationally, and clinically. An example of that is the gaps in medical professionals’ university training about rare diseases, which leads to them being insufficiently prepared to care for these patients and their families (Bokayeva et al., 2021; Walkowiak and Domaradzki, 2021). To reverse this situation in education, it is essential to raise the profile of these diseases (Jaffe et al., 2010; EURORDIS, 2011; Rath et al., 2012; Rodwell and Aymé, 2015) and develop multidimensional approaches to socio-educational and clinical actions (Monzón et al., 2017; Pérez et al., 2021).

These measures have become even more necessary in the face of the COVID-19 pandemic and its effects on society and schools (Chaabane et al., 2021; Lavidas et al., 2022). It has had a negative impact on the quality of life of children who need specific educational inclusion measures (Rodríguez and Gómez, 2021; Schwamberger, 2021), including those with rare diseases (González and González, 2021; Manuel-Guerra et al., 2021). There is a need for more education in order to properly meet this group’s needs (Wasilewska et al., 2022), an essential aspect to be analyzed in this study from the teaching perspective, and a key element in the students’ teaching and learning processes (Albrechtç et al., 2019).

The objective of this study was to investigate the possible influence of certain teaching variables on the teaching and learning processes of students with rare diseases. It aims to offer lines of action to consider for these students’ educational inclusion and to raise the possibility them being considered during initial and continuing teacher training for teachers in schools.

## 2. Materials and methods

The study was quantitative, through the collection of numerical data from the application of a questionnaire, and qualitative, through the observations and proposals for teaching improvement collected from the same questionnaire.

Both methodologies take a descriptive and interpretive approach to the educational reality of students with rare diseases in search of fairer and more inclusive education. The starting point is what teachers know and think about these students’ personal situations and education.

In this way, this study seeks to answer the following questions: What knowledge and perceptions do teachers have about students with rare diseases? Are there teaching sociodemographic variables that could influence this understanding?

### 2.1. Participants

The sample comprised 574 teachers from the Autonomous Community of Castilla-La Mancha, who teach non-university education, i.e., students aged between 3 and 18 years old, covering the following educational stages: Early Childhood Education, Primary Education, Compulsory Secondary Education, and Baccalaureate. They were selected by simple random sampling following initial contact with the schools’ management teams. The sociodemographic characteristics of the participants, and the frequency and percentages for each of the variables were as follows:

- Sex: Male (170 or 29.62%) or Female (404 or 70.38%). To specify this variable, we considered the proportion of teachers according to sex for this Spanish region (Ministerio de Educación y Formación Profesional, 2022).
- Age: 21–25 years old (15 or 2.61%), 26–35 years old (122 or 21.25%), 36–50 years old (296 or 51.57%), or 51 years and older (141 or 24.57%).
- Job position: Management Team (102 or 17.77%); Member of the Guidance and Support Team -EOA- in early childhood and primary schools, and special education schools/Guidance Department -DO- in secondary schools (116 or 20.21%); Home Room or Form Teacher (246 or 42.86%); or Other teaching category (110 or 19.16%).
- Years of teaching experience: 0–5 years (83 or 14.46%), 6–15 years (165 or 28.74%), 16–25 years (210 or 36.59%), or 26 years and over (116 or 20.21%).
- Higher academic qualifications: Grado (106 or 18.47%), Diplomatura (188 or 32.75%), Licenciatura (Bachelor’s degree; 186 or 32.40%), Master’s degree (83 or 14.46%), and Doctorate (11 or 1.92%).

### 2.2. Instrument

**Figure 1:**
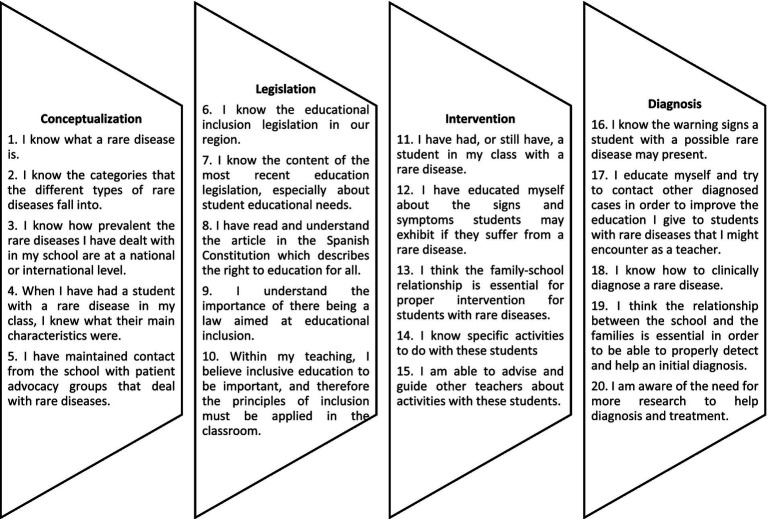
Structure of the questionnaire.

The instrument is made up of 20 items divided into four dimensions: Conceptualization or Dimension 1 (D1) includes knowledge covered by the term rare disease; Legislation or Dimension 2 (D2) covers the legal frameworks that determine the educational response toward these students (there are no exclusive regulatory frameworks for this group); Intervention or Dimension 3 (D3) covers specific actions in the classroom and aspects and contexts that influence their educational process; and Diagnosis or Dimension 4 (D4) composed of elements that have an impact on the processes of identifying these students. The questionnaire was structured as follows (Figure 1).

A Likert-type scale was used with the following responses and scores for each item: Not at all (1), A little (2), Somewhat (3), Quite a lot (4), and A lot (5). This means that the minimum score for each dimension is 5 and the maximum is 25. In addition to the items, the final part of the questionnaire is a section for observations and comments from the respondent to collect information in an open manner that the participants consider relevant to the study which may not have been covered by the individual items. In terms of statistical validation, the instrument demonstrated reliability indexes between 0.79 and 0.94 (Cronbach’s alpha was 0.94 for the total item score in the questionnaire and for each dimension it was: Conceptualization 0.80, Legislation 0.79, Intervention 0.85, and Diagnosis 0.79), content validity indexes were between 0.77 and 0.94 (overall content validity was 0.85 and for each dimension it was: Conceptualization 0.77, Legislation 0.94, Intervention 0.82, and Diagnosis 0.88), with a Kappa index of 0.88. Exploratory and confirmatory factorial analysis indicated a four-factor structure and a plausible fit for the initially designed model (exploratory factorial analysis: KMO index was 0.94, Bartlett’s sphericity test gave a value of 7699.08 and *p* < 0.001, and the percentage of total variance explained was 68.49%; confirmatory factorial analysis: CMIN = 32.48, *p* = 0.09, RMSEA = 0.05, CFI = 0.95, and TLI = 0.94), the factorial loads have been the following (Table 1).

**Table 1:** Factor loadings after exploratory factor analysis.

Items	Factor			
	1	2	3	4
IT1	0.745			
IT2	0.708			
IT3	0.711			
IT4	0.733			
IT5	0.610			
IT6			0.478	0.699
IT7				0.411
IT8		0.494		0.716
IT9				0.547
IT10				0.577
IT11		0.745		0.429
IT12		0.708		
IT13		0.711	0.428	
IT14		0.733		
IT15		0.610		
IT16			0.799	
IT17			0.694	
IT18			0.838	
IT19			0.818	
IT20			0.822	

### 2.3. Procedure and data analysis

The study took place between November and December 2021. First, a letter was sent to all school headteachers or directors in the region of Castilla-La Mancha indicating the content of the study asking them to tell their teachers about the study. The participating teachers gave their informed consent after being given all of the information related to the research process following approval from the corresponding Ethics Committee. All the information collected was kept confidential and was anonymous. Data analysis was *via* descriptive statistics, comparison of means, t-test, and ANOVA, using SPSS version 28. The original contributions presented in the study are included in the article/supplementary material, further inquiries can be directed to the corresponding author.

## 3. Results

Before presenting the analysis for each variable, the overall results from the questionnaire are shown in Table 2.

**Table 2:** Results for the questionnaire items and dimensions.

IT/D	Scores										M	SD
	1		2		3		4		5			
	*f*	%	*f*	%	*f*	%	*f*	%	*f*	%		
IT1	14	2.44	74	12.89	125	21.78	174	30.31	187	32.58	3.78	1.11
IT2	246	42.86	147	25.61	114	19.86	51	8.89	16	2.79	2.03	1.11
IT3	228	39.72	133	23.17	107	18.64	65	11.32	41	7.14	2.23	1.28
IT4	66	11.50	132	23.00	132	23.00	156	27.18	88	15.33	3.12	1.25
IT5	146	25.44	129	22.47	117	20.38	119	20.73	63	10.98	2.69	1.34
D1											13.85	4.74
IT6	34	5.92	43	7.49	118	20.56	157	27.35	222	38.68	3.85	1.19
IT7	118	20.56	80	13.94	97	16.90	134	23.34	145	25.26	3.19	1.47
IT8	18	3.14	39	6.79	95	16.55	150	26.13	272	47.39	4.08	1.09
IT9	6	1.05	9	1.57	57	9.93	135	23.52	367	63.94	4.48	0.82
IT10	8	1.39	14	2.44	51	8.89	147	25.61	354	61.67	4.44	0.86
D2											20.03	4.10
IT11	191	33.28	127	22.13	82	14.29	61	10.63	113	19.69	2.61	1.52
IT12	78	13.59	79	13.76	57	9.93	124	21.60	236	41.11	3.63	1.47
IT13	10	1.74	19	3.31	39	6.79	112	19.51	394	68.64	4.50	0.89
IT14	106	18.47	125	21.78	121	21.08	135	23.52	87	15.16	2.95	1.34
IT15	201	35.02	86	14.98	111	19.34	105	18.29	71	12.37	2.58	1.43
D3											16.27	5.40
IT16	160	27.87	146	25.44	130	22.65	107	18.64	31	5.40	2.48	1.23
IT17	106	18.47	127	22.13	121	21.08	144	25.09	76	13.24	2.93	1.32
IT18	278	48.43	126	21.95	103	17.94	45	7.84	22	3.83	1.97	1.15
IT19	24	4.18	41	7.14	53	9.23	113	19.69	343	59.76	4.24	1.14
IT20	7	1.22	9	1.57	18	3.14	68	11.85	472	82.23	4.72	0.71
D4											16.33	4.22
Total											66.49	16.41

As Table 2 shows, the highest mean scores were in Item 20 (M = 4.72, SD = 0.71), Item 13 (M = 4.50, SD = 0.89), and Item 9 (M = 4.48, SD = 0.82). The lowest scoring items were items 18 (M = 1.97, SD = 1.15), 2 (M = 2.03, SD = 1.11), and 3 (M = 2.23, SD = 1.28).

Of the four dimensions making up the questionnaire, legislation had the highest average results (M = 20.03, SD = 4.10), while conceptualization had the lowest (M = 13.85, SD = 4.74). These results were given more depth through examining the participating teachers’ observations and suggestions. In the Legislation dimension, the results indicate that teachers were aware of the regulatory frameworks that affect the educational inclusion of students with educational needs, they understood its importance, the need for greater legal support to promote patient support groups, the obligation that there is a regulated action and coordination protocol to be followed by all members of the educational community, and that there is a catalog of existing rare diseases that includes specific educational actions.

The results for the conceptualization dimension indicate the need to generalize broad training processes for teachers on this subject (characteristics and typology, diagnostic process, warning signs, and educational intervention), noting the importance of educational administrations in allocating resources—personnel and material—and the integration of external services to improve knowledge about these students’ characteristics and increase coordination between schools. This could result in more comprehensive, interdisciplinary multidimensional educational work with these students.

Finally, the overall mean was 66.49 (SD = 16.41), with asymmetry and negative or platykurtic kurtosis, −0.19 and − 0.82, respectively. Teachers stated that the breadth of existing rare diseases, the small numbers in schools, and the difficulties of generalizing specific educational actions from one disease to another—all of which have been reported by other studies (Nguengang-Wakap et al., 2020)—made it difficult for the scores from the questionnaire to be higher. These aspects hinder these students’ teaching and learning processes, but the question remains; are there other teacher-related variables that could have an impact on teachers’ knowledge and perceptions of rare diseases?

We attempt to answer this question by analyzing the results from the variables indicated above: sex, age, job position, years of teaching experience, and higher academic training achieved.

A comparison of means test based on the variable sex gave the following results (Table 3).

**Table 3:** T-test based on the sex variable.

D	Sex				*t*	df	*p*	*d*
	Male		Female					
	M	SD	M	SD				
D1	12.27	4.04	14.51	4.86	−5.30	572	<0.001^***^	0.48
D2	19.34	4.00	20.33	4.11	−2.67	572	0.008^**^	0.24
D3	14.62	5.35	16.97	5.28	−4.84	572	<0.001^***^	0.44
D4	15.02	4.11	16.89	4.14	−4.93	572	<0.001^***^	0.45
Total	61.25	15.35	68.70	16.35	−5.07	572	<0.001^***^	0.46

^**^Significant at 1% (*p* < 0.01). ^***^Significant at 0.01% (*p* < 0.001).

There were statistically significant differences in the four dimensions and in the total questionnaire score. Most of the differences had a significance *p* < 0.001. This is because the women scored higher than the men. The effect sizes were moderate (Cohen, 1988), ranging from 0.24 for the Legislation dimension to 0.48 for the conceptualization dimension.

The results with regard to teacher age were as follows (Table 4).

**Table 4:** ANOVA based on the variable teacher age (years).

D	Age								*F*	df	*p*	Eta^2^	Direction
	21–25 (1)		26–35 (2)		36–50 (3)		≥51 (4)						
	M	SD	M	SD	M	SD	M	SD					
D1	10.07	4.17	12.42	4.57	14.73	4.59	13.64	4.77	10.96	573	<0.001^***^	0.05	3 > 4 > 2 > 1
D2	20.40	2.90	18.91	4.59	20.65	3.83	19.68	4.08	5.81	573	<0.001^***^	0.03	3 > 1 > 4 > 2
D3	11.00	4.94	14.67	5.23	17.24	5.07	16.18	5.65	12.21	573	<0.001^***^	0.06	3 > 4 > 2 > 1
D4	13.60	4.64	13.60	4.64	17.09	3.78	16.11	4.42	9.43	573	<0.001^***^	0.05	3 > 4 > 2 > 1
Total	55.07	14.57	61.08	17.31	69.72	15.03	65.62	16.83	11.38	573	<0.001^***^	0.06	3 > 4 > 2 > 1

*** Significant at 0.01% (*p* < 0.001).

As Table 4 shows, there were statistically significant differences in the four dimensions and in the total questionnaire score, with most of the indices being *p* < 0.001. The highest scores were from teachers aged 36–50 in almost the entire questionnaire, followed by teachers aged 51 and over. The lowest general mean scores were from teachers aged 21–25, followed by teachers aged 26–35. Teacher age was key to the scores achieved in the questionnaire.

The results according to the professionals’ main work at their schools were as follows (Table 5).

**Table 5:** ANOVA based on the variable main job held.

D	Main job								*F*	df	*p*	Eta^2^	Direction
	Manag. team (1)		EOA/DO (2)		Tutor (3)		Other (4)						
	M	SD	M	SD	M	SD	M	SD					
D1	15.11	4.02	17.41	3.75	12.32	4.60	12.35	4.28	44.63	573	<0.001^***^	0.19	2 > 1 > 4 > 3
D2	21.63	3.22	23.46	1.98	18.46	3.95	18.46	3.95	66.58	573	<0.001^***^	0.26	2 > 1 > 3,4
D3	17.19	4.74	20.57	3.61	14.52	5.20	14.81	5.34	45.21	573	<0.001^***^	0.19	2 > 1 > 4 > 3
D4	17.27	2.91	19.71	2.91	14.93	4.26	15.05	4.09	48.67	573	<0.001^***^	0.20	2 > 1 > 4 > 3
Total	71.20	11.49	81.14	81.14	60.24	16.24	60.67	14.98	67.60	573	<0.001^***^	0.26	2 > 1 > 4 > 3

*** Significant at 0.01% (*p* < 0.001).

According to the ANOVA shown in Table 5, there were statistically significant differences in the instrument, all with a significance level of *p* < 0.001. Those working in guidance and support teams in early childhood and primary schools or in special education schools, and those in the guidance departments of secondary schools, had the best average scores in all parts of the questionnaire. The next best scores were from members of school management teams. The lowest scores were from home room teachers or from other professional profiles, the two teaching categories that spend the most time with a group of students throughout the school day. This gives us something to improve on in the delivery of inclusive processes, making it essential to generalize training activities in the field of rare diseases.

The results according to years of teaching experience were as follows (Table 6).

**Table 6:** ANOVA based on the teaching experience variable (years).

D	Teaching experience								*F*	df	*p*	Eta^2^	Direction
	0–5 (1)		6–15 (2)		16–25 (3)		≥ 26 (4)						
	M	SD	M	SD	M	SD	M	SD					
D1	14.13	4.63	13.34	4.73	14.27	4.65	13.62	4.97	1.37	573	0.251	0.01	3 > 1 > 4 > 2
D2	21.08	3.39	19.67	4.53	20.19	3.94	19.53	4.08	2.97	573	0.032^*^	0.01	1 > 3 > 2 > 4
D3	16.17	5.16	16.07	5.32	16.64	5.52	15.97	5.50	0.54	573	0.658	0.00	3 > 1 > 2 > 4
D4	17.10	4.00	15.87	4.44	16.70	3.97	15.79	4.37	2.77	573	0.041^*^	0.01	1 > 3 > 2 > 4
Total	68.48	14.89	64.95	17.33	67.80	15.94	64.91	16.77	1.70	573	0.166	0.01	1 > 3 > 2 > 4

* Significant at 5% (*p* < 0.05).

Table 6 indicates some statistically significant differences—although fewer than for the other variables—in the Legislation (*p* = 0.032) and Diagnosis (*p* = 0.041) dimensions. Teachers with 0–5 years of experience had higher mean scores.

Finally, the results in terms of academic qualifications were as follows (Table 7).

**Table 7:** ANOVA based on the variable highest academic qualification achieved.

D	Higher academic qualification										*F*	df	*p*	Eta^2^	Direction
	Grado (1)		Diplomatura (2)		Licenciatura (3)		Master (4)		Doctorate (5)						
	M	SD	M	SD	M	SD	M	SD	M	SD					
D1	11.63	4.38	13.91	4.88	14.33	4.55	15.02	4.37	17.27	4.56	9.52	573	<0.001^***^	0.06	5 > 4 > 3 > 2 > 1
D2	18.97	4.23	19.61	3.99	20.24	4.03	21.51	3.92	23.09	2.43	6.89	573	<0.001^***^	0.05	5 > 4 > 3 > 2 > 1
D3	13.73	5.37	16.32	5.40	16.75	5.11	17.87	5.06	19.91	4.48	9.89	573	<0.001^***^	0.06	5 > 4 > 3 > 2 > 1
D4	14.30	4.55	15.88	4.33	17.05	3.67	17.94	3.52	19.45	3.30	13.66	573	<0.001^***^	0.09	5 > 4 > 3 > 2 > 1
Total	58.63	17.05	65.72	16.38	68.37	15.29	72.34	14.19	79.73	13.17	12.08	573	<0.001^***^	0.08	5 > 4 > 3 > 2 > 1

*** Significant at 0.01% (*p* < 0.001).

As Table 7 shows, there were statistically significant differences in the four dimensions and in the total questionnaire score, all with a significance of *p* < 0.001. The highest scores were from teachers with doctoral degrees, followed by those with Master’s degrees. Teachers with the lower-ranking undergraduate degrees of Grado and Diplomatura had lower average scores.

## 4. Discussion

Social awareness and interest in the field of rare diseases has been consistently growing (Roessler et al., 2021) and is considered an emerging global public health priority (Nguengang-Wakap et al., 2020). In the educational field, inclusive practices are essential for schoolchildren with rare diseases, in order to increase their integration and quality of life (González and González, 2021; Wasilewska et al., 2022). This struggle to support and defend their quality of life is the objective of many national and international institutions (Centro de Referencia Estatal de Atención a Personas con Enfermedades Raras, 2022; Federación Española de Enfermedades Raras, 2022; Orphanet, 2022) that spread information and make suggestions to promote inclusion in the educational field. From the teaching perspective, teachers cannot tailor education solely from their professional experience; that must be complemented with specific training and close coordination with families and services outside the school, such as healthcare or patient support groups (García-Perales et al., 2022; Thombs et al., 2022).

Analyzing the results, the highest mean scores were for Item 20 (about the importance of dedicating more resources to research), Item 13 (on the need for family collaboration in educational intervention processes), and Item 9 (on the need to have legislation that strengthens the importance of inclusion in educational processes). Looking at the dimensions, the highest results were in Legislation. It could be said that teachers had higher scores in those items that demand more generic information about the educational response toward this group. For example, there is no regulatory framework enacted specifically for individuals with rare diseases, existing legislation is aimed generically at students with educational needs. In addition, it would be interesting to generalize the protocols and action guidelines for each of these rare diseases, preparing them using a collaborative approach combining educational, psychological, social, and clinical disciplines.

In contrast, there were lower mean scores for items requiring more specific information on rare diseases, such as Item 18 (on knowledge of the diagnostic process of a rare disease), Item 2 (on categorization), and Item 3 (related to prevalence). This was also the case when looking at the dimensions, since the lowest score was for the Conceptualization dimension. This indicates the need to extend specific training processes to better empower teachers to deliver tailored education to these children, being aware of the large number and variety of existing rare diseases. The relevant educational authorities would be essential for planning and carrying out these actions, and external services to schools that influence the educational work with these schoolchildren with rare diseases would also be key in raising the profile and awareness of their characteristics and needs (Nguengang-Wakap et al., 2020; García-Perales et al., 2022).

Turning to the teacher sociodemographic variables, we found that female teachers mostly had higher scores, with significance levels *p* < 0.001. This is in line with other studies that have concluded that women know more about and are more favorable toward students’ educational inclusion (Esteban-Guitart et al., 2012; Rodríguez-Fuentes and Caurcel-Cara, 2020; Falla et al., 2022), a pattern we also saw in the present study about rare diseases in all four dimensions of the questionnaire.

Teachers aged 36–50 had the highest mean scores, with indexes *p* < 0.001, in all dimensions and in the total mean score. There were also significant differences related to the type of job people did at their schools, with *p* < 0.001, the highest scores being from teachers who were part of school guidance services. This confirms how important these professionals are in delivering inclusive processes in schools (Nilsen et al., 2019; Sundqvist and Hannås, 2021). The amount of teaching experience was significant in two dimensions, Legislation and Diagnosis, with higher scores from teachers with 0–5 years’ teaching experience. This may be related to how close they were to the end of their degree courses and that they had maintained engagement and good attitudes toward educational inclusion (Álvarez-Castillo and Buenestado-Fernández, 2015; Falla et al., 2022). This could mean that they know more about—and are more aware of—the importance of an individualized educational response to students with rare diseases, in this case in aspects related to knowledge of legal regulations and identification of these schoolchildren.

Finally, there were statistically significant results in terms of academic qualifications; teachers with doctorates scored higher, most at *p* < 0.001. This confirms that academic training has an impact on the results of the questionnaire, the higher the qualifications, the higher the score. Teacher training is therefore a key variable in educational inclusion (García-González et al., 2018; Gutiérrez and Martínez, 2020). This is in line with what was previously stated for the Conceptualization dimension about the importance of training processes in the educational response to schoolchildren with rare diseases.

The results of this study may be used to increase awareness of and sensitivity toward rare diseases, for example, by increasing the generalization of training processes to improve teachers’ conceptualization of them. Education is an essential tool for students’ social and cultural inclusion, and by extension, teachers are an essential channel for achieving this end. As indicated in the theoretical framework, teachers’ attitudes toward inclusion are positive, a key aspect to take into consideration. Despite this, as we have seen, there are still outstanding tasks to be done to make progress toward the goal of having a fully inclusive social and educational system.

## 5. Conclusion

Teachers play a key role in the inclusion of students with rare diseases in all areas of their lives, hence its key consideration in this study. Teacher training and awareness have been shown to be fundamental in both educational intervention and in differential diagnosis. Knowledge of the specific characteristics of students is essential for individualizing their educational processes, which will help to raise the profile of these students’. That will make it possible to raise the profile of these students’ educational needs and thus increase their well-being. Much remains to be done and it is still a real challenge for educational, social and health systems to properly address those needs, something that other studies have also observed (Schieppati et al., 2008; Valdez et al., 2016; Nguengang-Wakap et al., 2020).

This study has some limitations: one is a lack of information about any prior training teachers may have had, another is the geographical location of the study as it only looked at one of the 17 Spanish autonomous communities. Future research will aim to replicate the results, considering the methodology used in this study and its limitations, including regression analysis between the variables. An additional future study proposal could include a research design to assess teachers’ knowledge and perceptions about other educational needs of students.

In short, despite the diversity and low prevalence of rare diseases in the educational field (Walker et al., 2017; Federación Española de Enfermedades Raras, 2018; Orphanet, 2022), this study has shown that there needs to be training and resources in order to be able to offer individualized educational responses to these students, an essential aspect of equitable, quality teaching, and learning processes. Educational authorities must ensure this, and make sure they have educational policies related to the inclusion of students with rare diseases.

## Data availability statement

The original contributions presented in the study are included in the article/supplementary material, further inquiries can be directed to the corresponding author.

## Ethics statement

The studies involving human participants were reviewed and approved by the University of Castilla-La Mancha. Participants provided written informed consent.

## Author contributions

RG-P and AP-R designed the study, collected, and analyzed the data, and wrote the manuscript. EG-T and MM-G contributed to the interpretation of the data and wrote, revised, and refined the manuscript. RG-P, AP-R, EG-T, and MM-G have participated in sending the article to the journal. All authors contributed to the article and approved the submitted version.

## Funding

This work was supported by the University of Castilla-La Mancha (UCLM).

## Conflict of interest

The authors declare that the research was conducted in the absence of any commercial or financial relationships that could be construed as a potential conflict of interest.

## Publisher’s note

All claims expressed in this article are solely those of the authors and do not necessarily represent those of their affiliated organizations, or those of the publisher, the editors and the reviewers. Any product that may be evaluated in this article, or claim that may be made by its manufacturer, is not guaranteed or endorsed by the publisher.
